# First study on the molecular prevalence of caprine arthritis encephalitis virus in goats in Babylon, Iraq

**DOI:** 10.14202/vetworld.2022.1129-1133

**Published:** 2022-04-28

**Authors:** Ahmed Hamzah Mosa, Karrar Jasim Hamzah, Hamed A. H. Aljabory

**Affiliations:** Department of Internal and Preventive Veterinary Medicine, College of Veterinary Medicine, AL-Qasim Green University, Babylon, Iraq

**Keywords:** caprine arthritis encephalitis virus, goats, Iraq, molecular detection, polymerase chain reaction

## Abstract

**Background and Aim:**

Caprine arthritis encephalitis virus (CAEV) is a virus that affects goats all over the world and causes enormous economic losses; as a result, screening for the disease is a priority, especially in Iraq. The present study aimed to estimate the prevalence of CAEV in infected goats using the précised PCR method in Babylon, Iraq.

**Materials and Methods:**

A total of 85 blood samples from goats aged 1 month to ≥6 years were analyzed for CAEV infections using molecular methods. The polymerase chain reaction primer was designed to amplify a 573 bp region of the proviral pol gene.

**Results:**

The CAEV tests revealed that five out of 85 goats were positive for CAEV. There were no significant differences in CAEV infection according to goat sex and significant differences according to age.

**Conclusion:**

Based on these results, the present study is the first molecular survey to confirm the current CAEV genome in an Iraqi goat flock.

## Introduction

Caprine arthritis encephalitis virus (CAEV) is a retrovirus that belongs to the lentivirus subfamily [1]. This virus causes mastitis, pneumonia, chronic progressive arthritis in adult goats, and leukoencephalomyelitis in young [2]. Domestic goats and sheep are infected throughout the world [3]. The high prevalence of this virus (80-95% in breeding stocks), especially in developing countries, is a source of concern [4]. There is evidence of direct transmission of CAEV infection by contact, body secretions, excretions, ingestion of virus-infected colostrum, goat milk, and goat to sheep or vice versa [5,6].

In terms of CAE prevalence, there are significant disparities in countries. Infection rates in Turkey and Mexico are as low as 1.9% and 3.6%, respectively, or as high as 82% in Australia and 23.2% in Jordan [6]. A study conducted in Iraq [7] discovered that the seroprevalence of CAEV infection is 8.69% in goats; they did not perform molecular surveys to diagnose the disease. The majority of the infected goats are asymptomatic, but some can express chronic clinical signs such as pneumonia, arthritis, and mastitis [2,8]. The vaccine or treatment for CAEV ineffective and supportive therapy is frequently costly [9,10]. CAEV infection has been linked to a significant decrease in productivity and causes economic losses [11]. As a result, its diagnosis must use a rapid, fast, and responsive process. Recent studies have shown that molecular methods such as polymerase chain reaction (PCR) offer high sensitivity, specificity, and precision for rapid CAEV identification in clinical samples [12,13]. These results highlight the survey importance of CAEV infections in the Middle East region, particularly in Iraq.

The present study aimed to estimate the prevalence of CAEV in infected goats using the précised PCR method in Babylon, Iraq.

## Materials and Methods

### Ethical approval

The present study was approved by the Ethical Committee of the Experimental Animal Production and Research Center of the College of Veterinary Medicine, AL-Qasim Green University, Babylon, Iraq, under protocol number 2020-77.

### Study period and location

The study was conducted from April 2020 to December 2020. Goats were selected from three different regions in Babylon of Iraq.

### Clinical examination

#### Clinical case history

The study includes 85 Iraqi goats (13 males and 72 females) with ages ranging from <2 years to more than 6 years. Temperature, pulse rate, respiratory rate, as well as detection of clinical signs such as emaciation, mastitis, arthritis, coughing, nervous signs, and dyspnea were recorded for each animal in a special format chart designed for these purposes.

### Isolation of peripheral blood mononuclear cells (PBMCs)

Each blood sample was diluted with 1:1 (volume) in sterile phosphate-buffered saline (PBS), pH 7.4, mixed, and then gently placed in a new sterile test tube containing an equal volume of lymphocyte separation medium (density gradient technique using Histopaque [1.077]). The samples were centrifuged for 20 min at 1000× *g*. The PBMCs appeared between Ficoll and plasma layers. A 1 mL micropipette was used to extract the separated PBMCs from the gradient interface into another sterile tube. PBMCs were separated and washed with PBS (2 mL), then mixed and centrifuged for 15 min at 700× *g*, twice repeated the washing pattern, resuspended, and kept at −20°C stored for PCR test [14].

### Total PBMCs genomic deoxyribonucleic acid (DNA) extraction

Genomic DNA samples from blood PBMCs were collected using the Genomic DNA Mini Extraction Kit (Geneaid, Taiwan) and done according to the manufacturer’s instructions. The extracted DNA was tested using a Nanodrop spectrophotometer (Thermo Fischer Scientific, United States), DNA concentration was measured (ng/μL) and read the absorbance at 260/280 nm to check the DNA purity.

### Primers

In this study, we used one PCR primer targeting pol genes; We designed primers using primer-blast software from National Center for Biotechnology Information’s (NCBI) GenBank database to investigate CAEV genes using the PCR technique.

Forward: 5’GTCTTTGCAGGCCACATTGG-3’

Reverse: 5’TGCCTTGCCTGATCCATGTT-3’

### PCR protocols

The extracted DNA used as a template to detect CAEV proviral DNA by single PCR was carried out using one set of pol primers only. We prepared the PCR master mix using the manufacturer’s instructions (AccuPower PCR PreMix Kit) and followed them exactly. The PCR master mix component was then placed in a typical standard AccuPower PCR PreMix Kit which contains all of the required components for PCR reaction such as Taq DNA polymerase, MgCl_2_, dNTPs, tracking dye, KCl, stabilizer, and Tris-HCl pH: 9.0. Furthermore, we centrifuged all the PCR tubes for 3 min at 3000 rpm in an ExiSpin vortex centrifuge. The samples were then placed in PCR Thermocycler (Techne, United States). The PCR pol gene products of CAEV were analyzed by 1.5 agarose gel electrophoresis. Amplified PCR products were obtained as 573 bp and visualized for pol gene using UV transilluminator.

### DNA sequencing and sequence analysis

Our isolates were also submitted to the NCBI to confirm the discovery of local CAEV and also to study the phylogenetic link between local CAEV and the NCBI-Blast database of CAEV. Positive samples by PCR targeting the pol gene of local isolates were sent by ice bag to the Macrogen Company in Korea for DNA sequencing analysis by the AB DNA sequencing system. Genome were sequenced and fastaq analysed using Mega 6 for aliment by ClustalW then phylogenetic constructed using the neighbor-joining method for phylogenetic tree analysis. The NCBI-BLAST was used to estimate the identity of the genetic sequence of local submission CAEV NCBI-Blast and some world isolates. The submission of NCBI-GenBank was performed using BankIt submission tool.

## Results

**Figure-1:**
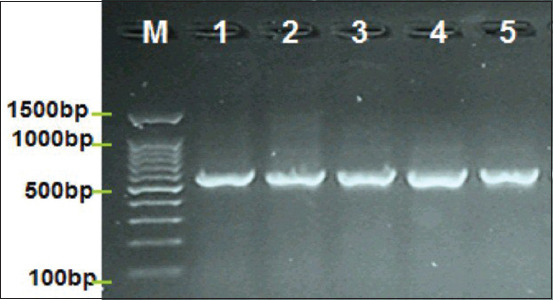
Image of 1.5 agarose gel electrophoresis reveals bands of PCR in positive CAEV isolates. Where: Lane (1-5) positive CAEV at 573 bp PCR product. PCR=Polymerase chain reaction, CAEV=Caprine arthritis encephalitis virus.

The detection of CAEV DNA in goats by PCR technique showed that five out of 85 samples (5.88%) were in Babylon/Iraq (Figure-1 and Table-1). In addition, the results showed that infection rates were not affected by gender (Table-1). Furthermore, Table-2 shows that the infection rates are nearly identical in male (2.08%) and female (5.22%)goats. Interestingly, our results showed higher infection rates in goats aged more 6 years (12%) than less 2 years (2.12%) Table-3.

**Table 1:** Infection rate of caprine arthritis encephalitis virus in goats according to PCR technique.

Governorates	Number of animals	Infected	Percentage
Babylon	85	5	5.88

PCR=Polymerase chain reaction

**Table 2:** Infection rates and gender relationship of blood samples examined by PCR technique.

Sex	Number of blood samples	Infected	Percentage
Males	13	1	7.69^A^
Females	72	4	5.55^A^
Total	85	5	5.88

Similar letters denote the non-significant differences at p*<*0.05. PCR=Polymerase chain reaction

**Table 3:** Infection rates and age relationship of blood samples examined by PCR technique.

Age/years	Number of blood samples	Infected	Percentage
<2	32	1	2.12^A^
2-6	28	1	3.57^A^
>6	25	3	12B
Total	85	5	5.88

Different letters denote the significant differences at p*>*0.05. PCR=Polymerase chain reaction

### Confirmation of PCR products by sequencing

The CAEV sequencing results were analyzed and annotated using the Basic Local Alignment Search Tool based on pol gene stored in the NCBI GenBank database.

### Phylogenetic analysis of CAEV

**Figure-2:**
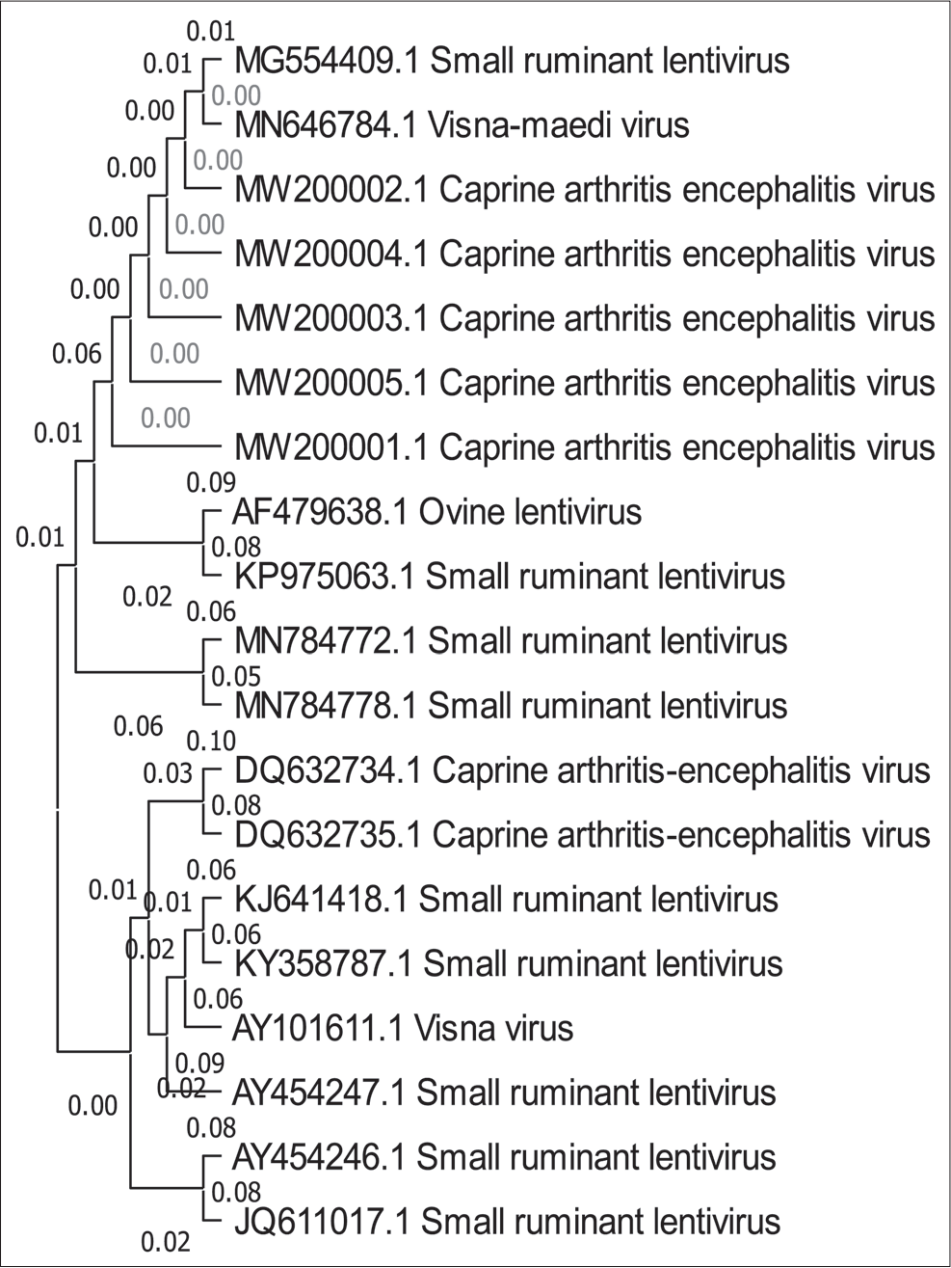
Phylogenetic tree of CAEV by maximum likelihood method based on partial sequence polymerase gene (pol) and relating the local strain of CAEV GenBank accession numbers sequence MW200001, MW200002, MW200003, MW200004, and MW200005 to other isolated strains. CAEV=Caprine arthritis encephalitis virus.

A simple phylogenetic tree based on partial polymerase gene sequences was generated to investigate the possible genetic relationship between CAEV strains detected in the present study and CAEV available in the GenBank database at the NCBI of polymerase gene (pol). The partial sequences of the pol gene of five positive CAEV samples (GenBank accession numbers MW200001, MW200002, MW200003, MW200004, and MW200005) were shown to be closely related to small ruminant lentivirus (SRLV), Visna-maedi virus, ovine lentivirus, and CAEV isolate found in GenBank (Table-4 and Figure-2).

**Table 4:** NCBI-BLAST homology sequence identity (%) for pol gene in local caprine arthritis encephalitis virus and NCBI-Blast isolates.

NCBI-BLAST isolate	Accession No.	Country	Host	NCBI-BLAST homology sequence identity (%)
SRLV	MG554409.1	Italy	Goat	100
Visna-maedi virus	MN646784.1	Iraq	Sheep	100
Ovine lentivirus	AF479638.1	Portugal	Sheep	86.34
SRLV	KY358787.1	USA	Sheep	84.82
SRLV	AY454247.1	Switzerland	Goat	84.26
SRLV	KP975063.1	Macedonia	Sheep	85.77
CAEV	DQ632734.1	Spain	Goat	83.65
Visna virus	AY101611.1	USA	Goat	83.62
CAEV	DQ632735.1	Spain	Goat	83.52
SRLV	JQ611018.1	Slovenia	Sheep	85.12
SRLV	AY454246.1	Switzerland	Goat	83.40
SRLV	MN784778.1	Belgium	Sheep	85.03
SRLV	JQ611018.1	Slovenia	Sheep	84.73
SRLV	MN784772.1	Belgium	Sheep	84.82
SRLV	JQ611017.1	Slovenia	Sheep	84.52

NCBI=National Center for Biotechnology Information, SRLV=Small ruminant lentivirus, CAEV=Caprine arthritis encephalitis virus

## Discussion

Using a specific primer targeting the pol gene, the PCR method is a gold standard and fast method for the detection of CAEV in PBMCs from infected goats [15,16]. DNA extracted from PBMCs was used as templates, with recorded sensitivities ranging from 70% to 95% [17,18]. In this study, the overall prevalence of infection detected by PCR technique is 5.88%; these results agreed with Jesse *et a*l. [4]. For the 1^st^ time in Iraqi goats, the presence of the CAEV genome in PBMCs has been confirmed. The current results showed that infection rates of CAEV in goats were lower than in Kosovo at 15.6% [19], Algeria at 29.7% [20], and Norway at 86% [21], but entirely higher than Honamli (1.60%) [22] and Italy 4.0% [3]. In a molecular investigation conducted on goats in Iran [23], the infection rate of CAEV was found to be 15.7%. Another study from Syria found that the prevalence of CAEV in the blood cells was 6.6% detected by PCR [24]. Furthermore, the results of Barták *et al*. [25] from the Czech Republic were higher than our results which revealed that the infection rate of CAEV in goats is 14.1%. Overall, the worldwide prevalence of CAEV has a large range of variation that could be as low as in Mexico at 3.6% and Iraq at 5.88% (current results) and high in Brazil at 75% [6]. Interaction with goats from other herds, purchased animals in herds, sheep on farms, and finally, herd size had all been identified as important risk factors for CAEV epidemiology and prevalence [26]. The infection rates in both sexes showed non-significant changes, and this was reported in Iraq [7] and Pakistan [27]. The goats over the age of 6 years had the highest infection rates of all age groups, while the goats under the age of 2 years had the lowest recorded rates. These results corroborated those of Mahmood *et al*. [27] and Arsenault *et al*. [28], who recorded that higher prevalence in adult animals, possibly due to chronic infection; this explained the chronicity character of the disease due to a long time of virus replication in the host macrophages and monocytes [29], in other words, this revealed the slow spread of disease in herds. Although seropositive cases were reported in <1 year of aged sheep [30], this is most likely due to the utero-infection [31]. Five CAEV local strains that have pol gene were also recorded under accession numbers: MW200001, MW200002, MW200003, MW200004, and MW200005 were analyzed in the constructed tree, revealed to be closely related to SRLV009 (MG554409.1), which is isolated in Italy. The phylogenetic tree was constructed from pol sequences revealed that the majority has affinities to CAEV-Co, on a separate branch from the Visna-Maedi group consisting of the SRLV Visna-Maedi virus and ovine lentivirus; this fact was also reported in the previous studies which showed evidence of transmission of disease from sheep to goat and out of trade livestock [8,12]. In France [32] and Italy [33], ovine isolates were determined to be similar to the caprine arthritis encephalitis prototype. SRLV (CAEV-Co) transmission from sheep to goats has been recorded in mixed herds as well as vice versa [34,35].

## Conclusion

The current research concluded that traditional PCR is a highly sensitive method for diagnosing CAEV in Babylon, Iraq. Future studies are needed with more than one set of primer for increasing diagnosis of CAEV and study the infection rate of CAEV in other breeds of goat in other governorate. In addition, the study of viral characteristics of Iraqi CAEV isolates should be conducted.

## Authors’ Contributions

AHM and KJH: Sample collection and laboratory work. HAHA: Analysis of the PCR results. AHM, KJH, and HAHA: Drafted and revised the manuscript. All authors have read and approved the final manuscript.
